# Calcium(II)_3_ (3,5-Diisopropylsalicylate)_6_(H_2_O)_6_ Activates Nitric Oxide Synthase: An Accounting for its Action in Decreasing Platelet Aggregation

**DOI:** 10.1155/MBD.2000.77

**Published:** 2000

**Authors:** Douglas C. Donham, John R. J. Sorenson

**Affiliations:** Division of Medicinal Chemistry, Department of Pharmaceutical Sciences College of Pharmacy University for Arkansas for Medical Sciences Campus Little Rock Arkansas 72205 USA

## Abstract

Purposes of these studies were first; to determine whether or not Calcium(II)_3_ (3,5- diisopropylsalicylate)_6_(H_2_O)_6_ [Ca(II)_3_(3,5-DIPS)_6_], a lipophilic calcium complex, could decrease activated-platelet aggregation, and second; to determine whether or not it is plausible that Ca(II)_3_(3,5-DIPS)_6_ decreases activated-platelet aggregation by facilitating the synthesis of Nitric Oxide (NO) by Nitric Oxide Synthase (NOS). The influence of Ca(II)_3_(3,5-DIPS)_6_ on the initial rate of activated-platelet aggregation was determined by measuring the decrease in rate of increase in transmission at 550 nm for a suspension of Thrombin-CaCl_2_ activated platelets following the addition of 0, 50, 100, 250, or 500 μM Ca(II)_3_(3,5-DIPS)_6_. To establish that the Ca(lI)_3_(3,5- DIPS)_6_-mediated decrease in aggregation was due to activation of NOS, the effect of ʟ-NMMA, an inhibitor of NOS, on the inhibition of platelet aggregation by Ca(II)_3_(3,5-DIPS)_6_ was determined using a suspension of activated platelets contaimng 0 or 250 μM Ca(II)_3_(3,5-DIPS)_6_ without or with 1 mM ʟ-NMMA. An *in vitro* Bovine Brain NOS reaction mixture, containing CaCl_2_ for the activation of Phosphodiesterase-3' ,5'-Cyclic Nucleotide Activator required for the activation of NOS, was used to determine whether or not Ca(II)_3_(3,5-DIPS)_6_ could be used as a substitute for the addition of Ca. The decrease in absorbance at 340 nm, lambda maximum for NADPH, was measured to determine NOS activity following the addition of NOS to the complete reaction mixture containing either CaCl_2_, Ca(II)_3_(3,5-DIPS)_6_, or neither Ca compound. Increasing the concentration of Ca(II)_3_(3,5-DIPS)_6_ caused a concentration related decrease in activated platelet aggregation. The addition of ʟ-NMMA to activated platelets, in the absence of Ca(II)_3_(3,5-DIPS)_6_, caused a 129% increase in initial rate of platelet aggregation. The initial rate of platelet aggregation decreased 74% with the addition of 250 μM Ca(II)_3_(3,5-DIPS)_6_ and the addition of ʟ-NMMA plus 250 μM Ca(II)_3_(3,5-DIPS)_6_ caused a 197% decrease in initial rate of aggregation compared to the initial rate observed width the presence of 1 mM ʟ-NMMA alone. There was only a small, 27%, increase in initial rate of 0.4 mM NADPH oxidation when 0.9 mM CaCl_2_ was added to the NOS reaction mixture in comparison to the initial rate of NADPH oxidation with no addition of CaCI_2_. Addition of an equivalent amount of Ca in the form of Ca(II)_3_(3,5-DIPS)_6_, 333 μM, caused a 37% increase in initial rate of NADPH oxidation compared to the addition of 0.9 mM CaCl_2_. Addition of increasing concentrations of ʟ-NMMA plus 0.9 mM CaCl_2_ or 333 μM Ca(II)_3_(3,5-DIPS)_6_ to the NOS reaction mixture caused a concentration related increase in initial rate of NADPH oxidation. Addition of ʟ-NMMA while expected to decrease NADPH oxidation actually increased the rate of NADPH oxidation. Additions of 133 μM or 267 μM Ca(II)_3_(3,5- DIPS)_6_ also caused concentration related increases in initial rate of NADPH oxidation in the presence of 113 μM ʟ-NMMA. However, the addition of 533 μM Ca(II)_3_(3,5-DIPS)_6_ caused a dramatic decrease in initial rate of NADPH oxidation by NOS. It is concluded that: 1) Ca(II)_3_(3,5- DIPS)_6_ activates platelet NOS in preventing platelet aggregation, 2) *in vitro* NOS activity can be observed spectrophotometrically by following the consumption of NADPH as a decrease in absorbance at 340 nm, 3) Ca(II)_3_(3,5-DIPS)_6_ plays a role in enhancing Bovine Brain NOS activity resulting in an increased rate of NADPH oxidation by NOS, 4) Ca(II)_3_(3,5-DIPS)_6_ is a useful form of Ca in activating NOS and superior to CaCl_2_ with regard to the facilitation of a NADPH oxidation, and 5) ʟ-NMMA stimulates Bovine Brain NOS activity rather than causing an inhibition of this enzyme and must serve as a reducible substrate for Bovine Brain NOS.


### CALCIUM(II)$_3$(3,5-DIISOPROPYLSALICYLATE)$_6$(H$_2$O)$_6$ ACTIVATES NITRIC OXIDE SYNTHASE: AN ACCOUNTING FOR ITS ACTION IN DECREASING PLATELET AGGREGATION

Douglas C. Donham and John R. J. Sorenson*

University for Arkansas for Medical Sciences Campus, Little Rock, Arkansas 72205, USA Division of Medicinal Chemistry, Department of Pharmaceutical Sciences, College of Pharmacy, e-mail: sorensonjohnr@exchange.uams.edu

Abstract

Purposes of these studies were first; to determine whether or not Calcium(II)$_3$(3,5-diisopropylsalicylate)$_6$(H$_2$O)$_6$ [Ca(II)$_3$(3,5-DIPS)$_6$], a lipophilic calcium complex, could decrease activated-platelet aggregation, and second; to determine whether or not it is plausible that Ca(II)$_3$(3,5-DIPS)$_6$ decreases activated-platelet aggregation by facilitating the synthesis of Nitric Oxide (NO) by Nitric Oxide Synthase (NOS). The influence of Ca(II)$_3$(3,5-DIPS)$_6$ on the initial rate of activated-platelet aggregation was determined by measuring the decrease in rate of increase in transmission at 550 nm for a suspension of Thrombin-$CaCl_2$ activated platelets following the addition of 0, 50, 100, 250, or 500 µM Ca(II)$_3$(3,5-DIPS)$_6$. To establish that the Ca(II)$_3$(3,5-DIPS)$_6$-mediated decrease in aggregation was due to activation of NOS, the effect of L-NMMA, an inhibitor of NOS, on the inhibition of platelet aggregation by Ca(II)$_3$(3,5-DIPS)$_6$ was determined using a suspension of activated platelets containing 0 or 250 µM Ca(II)$_3$(3,5-DIPS)$_6$ without or with 1 mM L-NMMA. An *in vitro* Bovine Brain NOS reaction mixture, containing $CaCl_2$ for the activation of Phosphodiesterase-3',5'-Cyclic Nucleotide Activator required for the activation of NOS, was used to determine whether or not Ca(II)$_3$(3,5-DIPS)$_6$ could be used as a substitute for the addition of Ca. The decrease in absorbance at 340 nm, lambda maximum for NADPH, was measured to determine NOS activity following the addition of NOS to the complete reaction mixture containing either $CaCl_2$, Ca(II)$_3$(3,5-DIPS)$_6$, or neither Ca compound. Increasing the concentration of Ca(II)$_3$(3,5-DIPS)$_6$ caused a concentration related decrease in activated platelet aggregation. The addition of L-NMMA to activated platelets, in the absence of Ca(II)$_3$(3,5-DIPS)$_6$, caused a 129% increase in initial rate of platelet aggregation. The initial rate of platelet aggregation decreased 74% with the addition of 250 µM Ca(II)$_3$(3,5-DIPS)$_6$ and the addition of L-NMMA plus 250 µM Ca(II)$_3$(3,5-DIPS)$_6$ caused a 197% decrease in initial rate of aggregation compared to the initial rate observed with the presence of 1 mM L-NMMA alone. There was only a small, 27%, increase in initial rate of 0.4 mM NADPH oxidation when 0.9 mM $CaCl_2$ was added to the NOS reaction mixture in comparison to the initial rate of NADPH oxidation with no addition of $CaCl_2$. Addition of an equivalent amount of Ca in the form of Ca(II)$_3$(3,5-DIPS)$_6$, 333 µM, caused a 37% increase in initial rate of NADPH oxidation compared to the addition of 0.9 mM $CaCl_2$. Addition of increasing concentrations of L-NMMA plus 0.9 mM $CaCl_2$ or 333 µM Ca(II)$_3$(3,5-DIPS)$_6$ to the NOS reaction mixture caused a concentration related increase in initial rate of NADPH oxidation. Addition of L-NMMA while expected to decrease NADPH oxidation actually increased the rate of NADPH oxidation. Additions of 133 µM or 267 µM Ca(II)$_3$(3,5-DIPS)$_6$ also caused concentration related increases in initial rate of NADPH oxidation in the presence of 113 µM L-NMMA. However, the addition of 533 µM Ca(II)$_3$(3,5-DIPS)$_6$ caused a dramatic decrease in initial rate of NADPH oxidation by NOS. It is concluded that: 1) Ca(II)$_3$(3,5-DIPS)$_6$ activates platelet NOS in preventing platelet aggregation, 2) *in vitro* NOS activity can be observed spectrophotometrically by following the consumption of NADPH as a decrease in absorbance at 340 nm, 3) Ca(II)$_3$(3,5-DIPS)$_6$ plays a role in enhancing Bovine Brain NOS activity resulting in an increased rate of NADPH oxidation by NOS, 4) Ca(II)$_3$(3,5-DIPS)$_6$ is a useful form of Ca in activating NOS and superior to $CaCl_2$ with regard to the facilitation of a NADPH oxidation, and 5) L-NMMA stimulates Bovine Brain NOS activity rather than causing an inhibition of this enzyme and must serve as a reducible substrate for Bovine Brain NOS.

### Introduction

Nitric Oxide (NO), synthesized by Nitric Oxide Synthase (NOS) mediates physiological responses including: neuronal transmission, consciousness, dilation of blood vessels, leukocyte destruction of invading pathogens and cancer cells, leukocyte responses in inflammation, pain perception, and decreased platelet adhesion and aggregation [1,2 and references therein].

Nitric Oxide and L-Citrulline are formed via a reduced nicotinamide-adenine-dinucleotide-3'-phosphate (NADPH)-dependent enzymatic oxidation of L-Arginine by NOS. There are two forms of NOS. The constitutive form of NOS is reversibly activated by the Ca-dependent Phosphodiesterase-3',5'-Cyclic Nucleotide Activator known as Calmodulin, which is activated by an influx of Ca into the cells or mobilization of stored Ca. The inducible form of this enzyme is activated by white blood cell derived cytokines [1].

Cellular uptake of Ca is actively regulated in part by NO activation of Guanylyl Cyclase, the subsequent synthesis of cyclic guanosine monophosphate, and the release of Glutamic acid which in turn opens Ca channels allowing Ca to enter cells, enabling the activation of Calmodulin. The form of Ca entering platelets is generally depicted as hydrophillic, non-lipophilic, ionically bonded Ca [3].

Since a more lipophilic form of Ca may be able to undergo translocation across platelet membranes in a passive process, Calcium(II)$_3$(3,5-diisopropylsalicylate)$_6$($H_2O$)$_6$ [Ca(II)$_3$(3,5-DIPS)$_6$], a lipophilic Ca complex, was examined for its effects on platelet aggregation. Platelet aggregation was studied in an activated-platelet system containing 1 mM ionically bonded Ca, in the form of $CaCl_2$, and Thrombin, both being required for platelet aggregation [4].

The monomethyl derivative of L-Arginine, N$^G$-monomethyL-L-Arginine (L-NMMA), is suggested to inhibit NOS following bonding to the active oxidation domain of NOS and prevent the production of NO causing an increase in platelet aggregation [2,4]. This inhibitor of NOS was viewed as useful in determining whether or not Ca(II)$_3$(3,5-DIPS)$_6$ inhibits platelet aggregation by activation of NOS and determining whether or not L-NMMA inhibits the Ca(II)$_3$(3,5-DIPS)$_6$-mediated inhibition of platelet aggregation.

Materials and Methods

Tetrahydrobiopterin (THB), NADPH, L-NMMA, Calmodulin, D, L-Dithiothreitol (DTT), flavin adenine dinucleotide (FAD), L-Arginine, Bovine Brain NOS, Thrombin, $CaCl_2$ (all from Sigma Chemical Company), 4-(2-hydroxyethyl)-1-piperazinethane-sulfonic acid (HEPES) (Aldrich Chemical Company) and 3,5-diisopropylsalicylic acid (Hochem Enterprise) were used as purchased without further purification.

Calcium(II)$_3$(3,5-DIPS)$_6$ was synthesized as follows. One hundred grams (0.45 mol) of 3,5-diisopropylsalicylic acid was dissolved in two liters of deionized water with 29.7 g (0.45 mol) of KOH with vigorous stirring. The resulting solution was adjusted to pH 9.0 with 10% hydrochloric acid and filtered with a sintered glass funnel. A solution of $CaCl_2$ was prepared by dissolving 36.39 g (0.50 mol) $CaCl_2(H_2O)_2$, a 10% excess of Ca, in 500 ml of deionized water and filtered through a sintered glass funnel. The $CaCl_2$ solution, pH 7.3, was added dropwise with a separatory funnel to a vigorously stirred solution of potassium 3,5 diisopropylsalicylate. Vigorous stirring was employed to cause shearing of the precipitating Ca complex particles and avoid entrapment of the reactant and potassium chloride in the precipitating reaction product. Following completion of this addition, the product was collected by filtration with a sintered glass funnel and air dried overnight in the filter funnel attached to laboratory vacuum, 15 mm Hg. A 78% yield, 84.7 g, of a white solid melting over the range of 240° to 243°C was obtained using these procedures. X-ray crystallography revealed that the complex is a linear trinuclear hexahydrate, Ca(II)$_3$(3,5-DIPS)$_6$($H_2O$)$_6$, hexakis-μ-3,5-diisopropylsalicylatohexaaquotricalcium(II) [Professor Wally Cordes, personal communication]. Elemental composition calculated for $C_{78}H_{114}O_{24}Ca_3$: C, 60.24% and H, 7.33%. Found values were: C, 60.23% and H, 7.04%.

Rat platelets were collected prior to their use on the day they were used and maintained in a platelet buffer containing HEPES, Sodium Chloride, Potassium Chloride, Glucose, and Indomethacin, added to inhibit cyclooxygenase activity. Ultraviolet and visible absorbances were measured with a Hewlett Packard 8452A Diode Array Spectrophotometer. All glassware was thoroughly cleaned with either aqua regia or Citronox (Alconox Inc.) and all plasticware was metaL-free polypropylene. Deionized water, pH 7.5, was used throughout.

*Platelet Inhibition Studies*: A 100 mM Ca(II)$_3$(3,5-DIPS)$_6$ solution was prepared by dissolving 1.55 g (1 mmol) in enough absolute ethanol to make the final volume 10 ml. Approximately 12 ml of rat blood was centrifuged at 150 x g for 20 minutes. The separated plasma was then centrifuged at 800 x g for 15 minutes to obtain a platelet pellet. A stock suspension of platelets was prepared by adding 10 ml of buffer to the platelet pellet and gently shaking the solution. A stirring magnet, 1 ml of buffer, 500 μl of platelet suspension, 7.5 μl of ethanol, and 37.5 μl of 250 μM $CaCl_2$ were placed in a 4 ml cuvette. Using a Hitachi spectrophotometer, the percent transmission was recorded at 550 nm for two minutes. One hundred micro units of Thrombin (15 μl) was then added five seconds after the recording of percent transmission began. These spectrophotometric recordings were repeated with the replacement of the 7.5 μl of ethanol with 7.5 μl of 2, 10, 20, 50, or 100 mM ethanolic $Ca(II)_3(3,5\text{-}DIPS)_6$ to give a final concentration of 10, 50, 100, 250, or 500 μM $Ca(II)_3(3,5\text{-}DIPS)_6$. Platelets were also incubated with 1 mM L-NMMA for 5 minutes or with 250 μM $Ca(II)_3(3,5\text{-}DIPS)_6$ and 1 mM L-NMMA and percent transmission determined over a period of two minutes to determine effects of these two compounds on the rate of activated platelet aggregation. Data are presented as the average of 3 to 5 determinations following the addition of the examined compounds.

The rate of aggregation of activated platelets was measured as the time-dependent increase in transmission of incident irradiation. The initial rate of aggregation was determined by measuring the increase in transmission per second. Percent inhibition of the rate was calculated by subtracting the observed initial rate of aggregation for the activated platelet reaction mixture containing no $Ca(II)_3(3,5\text{-}DIPS)_6$ from the observed initial rate of aggregation of activated platelets for the reaction mixture containing $Ca(II)_3(3,5\text{-}DIPS)_6$, dividing by the initial rate of aggregation for the activated platelet reaction mixture containing no $Ca(II)_3(3,5\text{-}DIPS)_6$, and multiplying by 100.

*In Vitro Studies of Nitric Oxide Synthase*: An 80 mM HEPES buffer (pH 7.3) containing 1.5 mM $CaCl_2$ ($CaCl_2$ Buffer) was prepared by dissolving 19.064 g of HEPES and 0.2205 g of $CaCl_2$ in enough deionized water to make the final volume one liter. To prepare a 3.4 mM L-Arginine solution, 7.164 mg (34 μmol) was dissolved in enough $CaCl_2$ Buffer to make the final volume 10 ml. An 80 mM HEPES buffer (pH 7.3) containing no Ca (Buffer) was prepared by dissolving 19.064 g (80 mmol) of HEPES in enough deionized water to make the final volume one liter. A 1 mM $Ca(II)_3(3,5\text{-}DIPS)_6$ solution was prepared by dissolving 1.55 g (10 mmol) of $Ca(II)_3(3,5\text{-}DIPS)_6(H_2O)_6$ in enough Buffer to make the final volume 10 ml. To prepare a 20 mM DTT solution, 18.02 mg (0.2 mmol) was dissolved in enough Buffer to make the final volume 5 ml. To prepare a 1 mM THB solution, 3.142 mg (10 μmol) was dissolved in enough Buffer to make the final volume 10 ml. This solution was made immediately before use. To prepare a 1.0 mM FAD solution, 2.295 mg (10 μmol) was dissolved in enough Buffer to make the final volume 10 ml. To prepare a 3.0 mM NADPH solution, 2.5 mg (30 μmol) was dissolved in enough Buffer to make the final volume 10 ml. To prepare a 5000 unit/ml Calmodulin solution, 10,000 units of Calmodulin were dissolved in two ml of Buffer. A 3.4 mM L-NMMA solution was prepared by dissolving 3.38 mg (17 μmol) of L-NMMA in enough Buffer to make the final volume 5 ml.

Using the Hewlett Packard 8452A Diode Array spectrophotometer with a cuvette magnetic stirrer, spectra of DTT, THB, FAD, L-NMMA, L-Arginine, $Ca(II)_3(3,5\text{-}DIPS)_6$, and NADPH were recorded throughout the range of 200 nm to 800 nm. One tenth of a ml of a 5000 unit/ml Calmodulin, 0.9 ml of Buffer, 0.1 ml of the DTT solution, 0.1 ml of the 1 mM THB solution, and 0.025 ml of the 1 mM FAD solution were added to the cuvette, stirred, and maintained at 37°C. Then, 50 μl of the 3.4 mM L-Arginine solution, 200 μl of the 3 mM NADPH solution, and 25 μl of NOS were added to the cuvette. The change in absorbance at 340 nm was recorded every minute for fifteen minutes after the addition of NOS. This experiment was repeated using 0.9 ml of $CaCl_2$ Buffer in place of the Buffer lacking $CaCl_2$.

To measure the effect of $Ca(II)_3(3,5\text{-}DIPS)_6$ on NOS, 0.1 ml of 5000 unit/ml Calmodulin, 0.4 ml of Buffer, 0.5 ml of the 1 mM $Ca(II)_3(3,5\text{-}DIPS)_6$ solution, 0.1 ml of the DTT solution, 0.1 ml of the 1 mM THB solution, and 0.025 ml of the 1 mM FAD solution were added to the cuvette and stirred at 37°C. Then, 50 μl of the 3.4 mM L-Arginine solution, 200 μl of the 3.0 mM NADPH solution, and 25 μl of NOS were added to the cuvette. The change in absorbance at 340 nm was recorded every minute for fifteen minutes after the addition of NOS.

To measure the effect of the NOS inhibitor L-NMMA on the NOS enzyme system, 0.1 ml of a 5000 unit/ml Calmodulin, 0.85 ml of $CaCl_2$ Buffer, 0.1 ml of the DTT solution, 0.1 ml of the 1 mM THB solution, and 0.025 ml of the 1 mM FAD solution were added to the cuvette and stirred at 37°C. Then, 50 μl of the 3.4 mM L-Arginine solution, 50 μl of the 3.4 mM L-NMMA solution, 200 μl of the 3 mM NADPH solution, and 25 μl of NOS were added to the cuvette. The change in absorbance at 340 nm was recorded every minute for fifteen minutes after the addition of NOS. This experiment was repeated by replacing 200 μl or 400 μl of $CaCl_2$ Buffer with 200 μl or 400 μl of L-NMMA solution.

To determine the effect of L-NMMA on NOS in the presence of $Ca(II)_3(3,5\text{-}DIPS)_6$, 0.1 ml of 5000 unit/ml Calmodulin, 0.5 ml of the 1 mM $Ca(II)_3(3,5\text{-}DIPS)_6$ solution, 0.1 ml of the DTT solution, 0.1 ml of the 1 mM THB solution, and 0.025 ml of the 1 mM FAD solution were added to the cuvette and stirred at 37°C. Then, 50 μl of the 3.4 mM L-Arginine solution, 40 μl of the 3.4 mM L-NMMA solution, 200 μl of the 3.0 mM NADPH solution, and 25 μl of NOS were added to the cuvette. The change in absorbance at 340 nm was recorded every minute for fifteen minutes after the addition of NOS. This experiment was repeated using 0.05 ml of 3.4 mM L-NMMA and 0.2 ml, 0.4 ml, 0.5 ml, or 0.8 ml of 1 mM $Ca(II)_3(3,5\text{-}DIPS)_6$ solution and enough Buffer to make the final volume 1.5 ml. For each experiment involving the addition of $CaCl_2$, $Ca(II)_3(3,5\text{-}DIPS)_6$, and/or L-NMMA to the NOS reaction mixture the decrease in absorbance at 340 nm was plotted over a 15 minute period. To obtain the initial rate of NADPH oxidation, the decrease in 340 nm absorbance observed over the first five minutes was used to obtain a regression plot at the 95% confidence level using Sigma Plot (Jandel Corporation). Other statistics including $r^2$, the goodness of fit, and m, the slope, were also obtained at the 95% confidence level with the Sigma Plot program. These regression plots were used to calculate the initial rate of the enzyme catalyzed reaction using the formula: $\Delta A\ min^{-1} \times 100$.

Results

Initial studies demonstrated that the use of ethanol to make additions of Ca(II)$_3$(3,5-DIPS)$_6$ to the reaction mixture had no effect on aggregation of platelets activated with the addition of 1 μl $CaCl_2$ plus 100 μU of Thrombin. The initial rate for the increase in percent transmission of the 600 nm incident irradiation, associated with aggregation of platelets, following the addition of 75 μl of ethanol was 0.7 percent transmission/second (%T/s) versus 0.6 %T/s for the aqueous system containing no ethanol.

In addition, Ca(II)$_3$(3,5-DIPS)$_6$ did not serve as a more bioavailable source of Ca when compared to $CaCl_2$ in enabling Thrombin activation of platelets. The initial rate of aggregation of activated platelets in the presence of 1 μM $CaCl_2$ was 0.1 %T/s versus 0.15 %T/s when up to 500 μM Ca(II)$_3$(3,5-DIPS)$_6$ was added to the reaction mixture instead of $CaCl_2$. This addition of Ca(II)$_3$(3,5-DIPS)$_6$ represented a 1500 fold increase in the amount of Ca supplied by the addition of 1 μM $CaCl_2$ required to activate platelets.

Increasing the concentration of Ca(II)$_3$(3,5-DIPS)$_6$ added to the Thrombin and $CaCl_2$ activated platelet system caused a concentrated related decrease in platelet aggregation, as shown in Figure 1 and Table I. Consistent with this, the percent inhibition of the initial rate of platelet aggregation increases as the concentration of Ca(II)$_3$(3,5-DIPS)$_6$ increased as shown in Table 1.

**Figure 1. Initial rate of aggregation following the addition of Ca(II)$_3$(3,5-DIPS)$_6$ to activated platelets.**

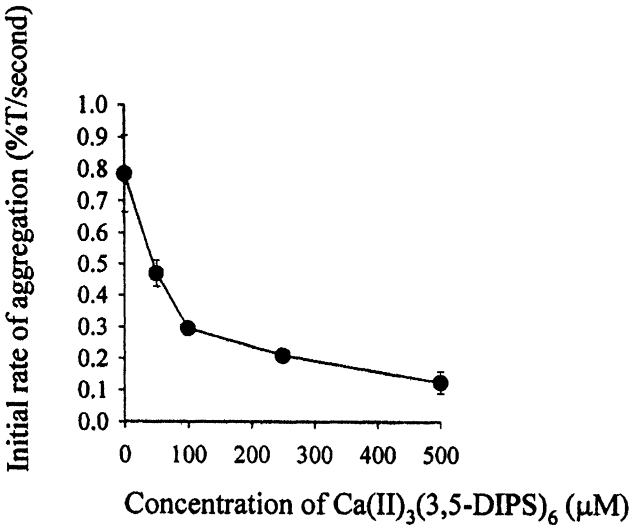

Table I. Initial rate of platelet aggregation and percent inhibition in the presence of increasing concentrations of Ca(II)$_3$(3,5-DIPS)$_6$.

| Concentration of Ca(II)$_3$(3,5-DIPS)$_6$ (μM) | Initial Rate of Aggregation (% increase in transmission/sec) | Percent Inhibition |
|---|---|---|
| 0 | 0.87 | 0 |
| 50 | 0.44 | 50 |
| 100 | 0.29 | 67 |
| 250 | 0.22 | 75 |
| 500 | 0.15 | 83 |

As shown in Figure 2 and Table II, additions of $Ca(II)_3(3,5\text{-}DIPS)_6$ caused a decrease in platelet aggregation in the presence or absence of L-NMMA. The initial rate of aggregation for $CaCl_2$ and Thrombin activated platelets was 0.31 %T/s. Addition of 250 μM $Ca(II)_3(3,5\text{-}DIPS)_6$ to the reaction mixture decreased the initial rate of aggregation of these platelets to 0.08 %T/s, nearly a four-fold decrease in rate of aggregation. Incubation of activated platelets with 1 mM L-NMMA increased the rate of aggregation to 0.71 %T/s, a two-fold increase in rate of aggregation. Addition of both 250 μM $Ca(II)_3(3,5\text{-}DIPS)_6$ and 1 mM L-NMMA caused a reduction of the initial rate of aggregation to 0.10 %T/s, a seven-fold decrease in aggregation compared to the aggregation observed for the addition of L-NMMA alone. The percent decrease in rate of aggregation, shown in Table 2, reveals that $Ca(II)_3(3,5\text{-}DIPS)_6$ alone prevents aggregation and enhanced aggregation due to the addition of L-NMMA was also prevented by the addition of $Ca(II)_3(3,5\text{-}DIPS)_6$ (Table 2).

**Figure 2. Effect $Ca(II)_3(3,5\text{-}DIPS)_6$(--) or $Ca(II)_3(3,5\text{-}DIPS)_6$ plus L-NMMA (–) addition on the initial rate of platelet aggregation.**

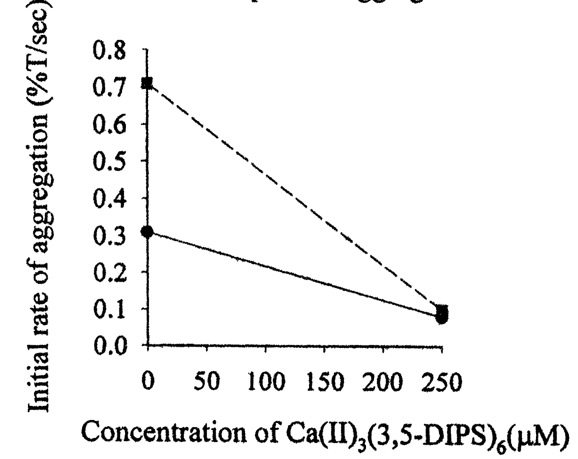

Table II. Effect of the addition of $Ca(II)_3(3,5\text{-}DIPS)_6$ or $Ca(II)_3(3,5\text{-}DIPS)_6$ plus L-NMMA on the initial rate of platelet aggregation.

| Concentration of $Ca(II)_3(3,5\text{-}DIPS)_6$ (μM) | Initial Rate of Aggregation (% increase in transmission/sec) | Percent Inhibition |
|---|---|---|
| 0 | 0.31 | 0 |
| 250 | 0.08 | 74 |
| 0 + L-NMMA | 0.71 | -129 |
| 250 + L-NMMA | 0.10 | 68 |

Nitric Oxide Synthase activity was determined by measuring the decrease in 340 nm absorbance for the oxidation of NADPH by NOS in the conversion of L-Arginine to L-Citrulline and NO. The addition of NOS to the incomplete reaction mixture containing no added Ca caused the oxidation of NADPH as shown in Figure 3. The initial rate for this oxidation, 0.22 nm/min, was calculated, $\Delta A\ min^{-1} \times 100$, using the regression plot (Figure 3a) for data obtained during the first five minutes of NADPH oxidation. As shown in Figure 3a, data obtained over this period were linear and had a good fit to this regression plot ($r^2 = 0.98$).

**Figure 3. Oxidation of 0.4 mM NADPH by Nitric Oxide Synthase in the absence of 0.9 mM $CaCl_2$.**

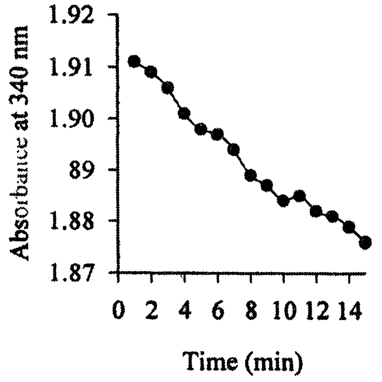

**Figure 3a. Initial rate for the oxidation of 0.4 mM NADPH by Nitric Oxide Synthase in the absence of 0.9 mM $CaCl_2$.**

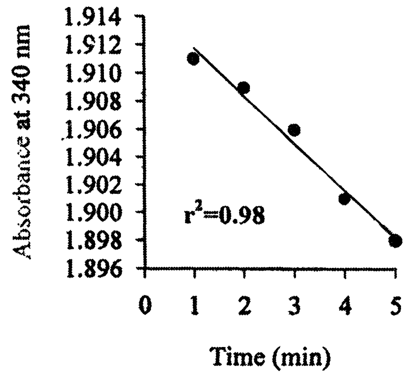

The addition of NOS to the complete reaction mixture containing 0.9 mM $CaCl_2$ caused the oxidation of NADPH shown in Figure 4, with an initial rate of 0.48 nm/min calculated using data presented in Figure 4a. Table III is a summary of the initial rates of oxidation of NADPH by NOS, the decrease in absorbance at 340 nm during the first five minutes for each of these experiments. There was a 27% increase in initial rate with the addition of $CaCl_2$ compared to the incomplete reaction mixture containing no added $CaCl_2$.

Table III. Initial rate of 400 μM NADPH oxidation by Nitric Oxide Synthase.

| Addition or Deletion to the Reaction Mixture | Initial Rate of NADPH Oxidation (decrease in A/min x 100) | Percent Change in Initial Rate versus System with no $CaCl_2$ |
|---|---|---|
| No added $CaCl_2$ | 0.26* | ---- |
| 0.9 mM $CaCl_2$ | 0.33* | 27 |
| 333 μM Ca(II)$_3$(3,5-DIPS)$_6$ | 0.45 | 73 |
| 0.9 mM $CaCl_2$ and 113 μM L-NMMA | 0.45 | 73 |
| 0.9 mM $CaCl_2$ and 453 μM L-NMMA | 0.73 | 181 |
| 0.9 mM $CaCl_2$and 907 μM L-NMMA | 0.80 | 208 |
| 333 μM Ca(II)$_3$(3,5-DIPS)$_6$ and 907 μM L-NMMA | 1.13 | 335 |
| 133 μM Ca(II)$_3$(3,5-DIPS)$_6$ and 113 μM L-NMMA | 0.88 | 239 |
| 267 μM Ca(II)$_3$(3,5-DIPS)$_6$ and 113 μM L-NMMA | 1.10 | 323 |
| 533 μM Ca(II)$_3$(3,5-DIPS)$_6$and 113 μM L-NMMA | 0.19 | -27 |

*Average of 3 to 5 determinations

Addition of 333 μM Ca(II)$_3$(3,5-DIPS)$_6$ increased the initial rate of NADPH oxidation (Figures 5 and 5a) to 73% (Table III) compared to the incomplete reaction mixture containing no added $CaCl_2$

Figure 4. Oxidation of 0.4 mM NADPH by Nitric Oxide Synthase in the presence of 0.9 mM $CaCl_2$.

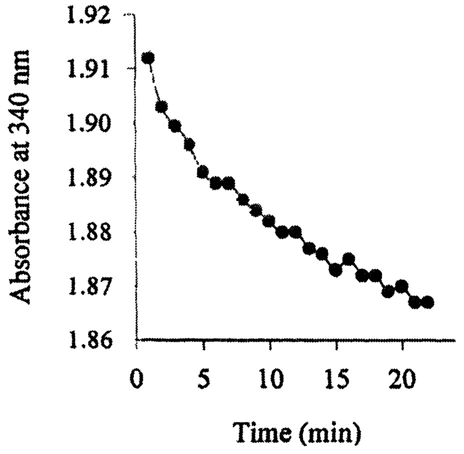

Figure 4a. Initial rate for the oxidation of 0.4 mM NADPH by Nitric Oxide Synthase in the presence of 0.9 mM $CaCl_2$.

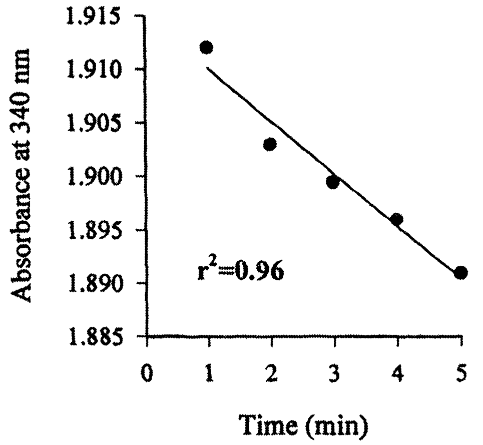

Figure 5. Oxidation of 0.4 mM NADPH by Nitric Oxide Synthase in the presence of 333 μM $Ca(II)_3(3,5\text{-DIPS})_6$.

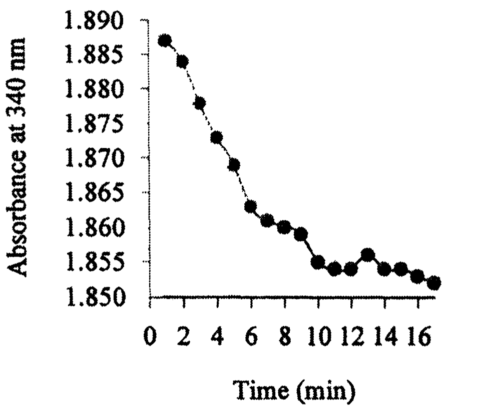

Figure 5a. Initial rate for the oxidation of 0.4 mM NADPH by Nitric Oxide Synthase in the presence of 333 μM $Ca(II)_3(3,5\text{-DIPS})_6$.

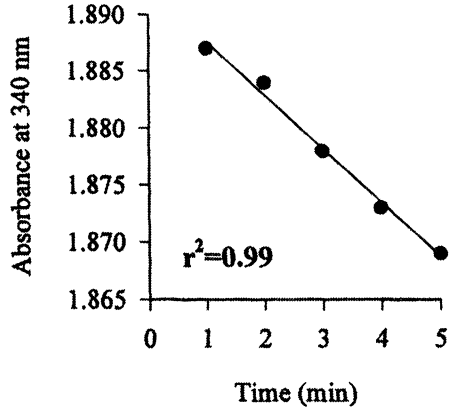

Figure 6. Oxidation of 0.4 mM NADPH by Nitric Oxide Synthase in the presence of 0.9 mM $CaCl_2$ and 113 μM L-NMMA.

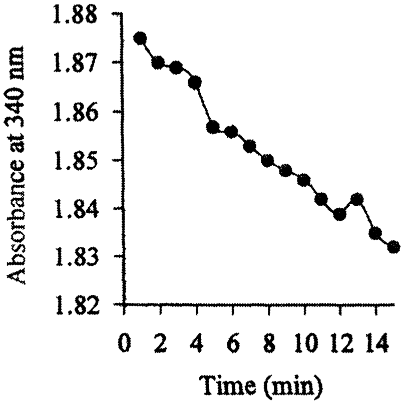

Figure 6a. Initial rate for the oxidation of 0.4 mM NADPH by Nitric Oxide Synthase in the presence of 0.9 mM $CaCl_2$ and 113 μM L-NMMA.

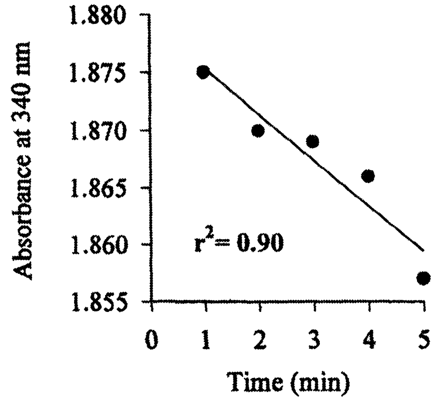

Adding 113 μM L-NMMA to the complete reaction mixture containing $CaCl_2$ also increased the initial rate of NADPH oxidation (Figures 6 and 6a) to 73 % (Table III) in comparison with the initial rate observed for the incomplete reaction mixture.

As shown in Table III the addition of 453 μM L-NMMA to the complete reaction mixture containing $CaCl_2$ caused a further linear increase in initial rate of NADPH oxidation (Figures 7 and 7a) to 181 %.

Figure 7. Oxidation of 0.4 mM NADPH by Nitric Oxide Synthase in the presence of 0.9 mM $CaCl_2$ and 453 μM L-NMMA.

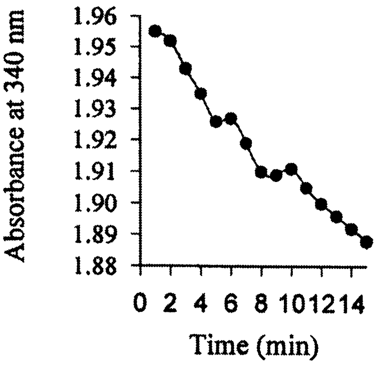

Figure 7a. Initial rate for the oxidation of 0.4 mM NADPH by Nitric Oxide Synthase In the Presence of 0.9 μM $CaCl_2$ and 453 μM L-NMMA.

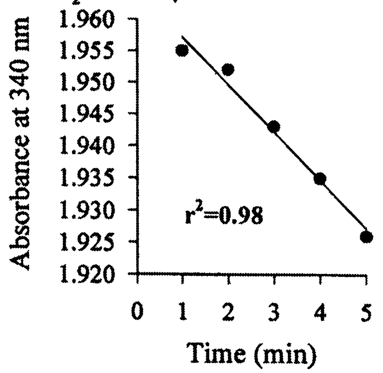

With the addition of 907 μM L-NMMA, there was a further linear (Figures 8 and 8a) increase in initial rate of oxidation of NADPH to 208% (Table III).

Figure 8. Oxidation of 0.4 mM NADPH by Nitric Oxide Synthase in the presence of 0.9 mM $CaCl_2$ and 907 μM L-NMMA.

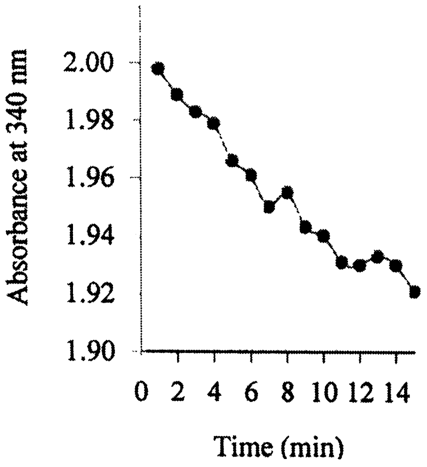

Figure 8a. Initial rate for the oxidation of 0.4 mM NADPH by Nitric Oxide Synthase in the presence of 0.9 mM $CaCl_2$ and 907 μM L-NMMA.

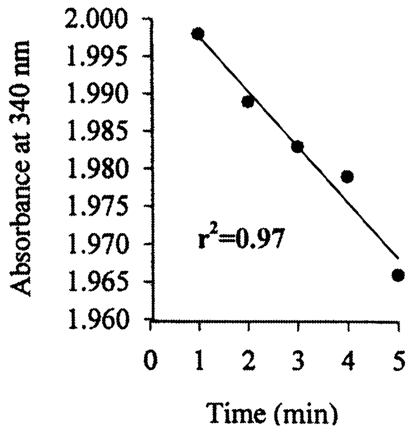

Replacing the 0.9 mM $CaCl_2$ with 333 μM Ca(II)$_3$(3,5-DIPS)$_6$ and adding 907 μM L-NMMA caused a still greater increase in initial rate of NADPH oxidation (Figures 9 and 9a) to 335% (Table III) compared to the reaction mixture without 0.9 mM $CaCl_2$ and 127 % greater than the reaction mixture containing 0.9 mM $CaCl_2$ and 907 μM L-NMMA. However the time-dependent decrease in absorbance was not as linear over the 15 min. period of measurement. Increasing the concentration of Ca(II)$_3$(3,5-DIPS)$_6$ from 133 μM to 267 μM and holding the concentration of L-NMMA at 113 μM caused further concentration related increases in initial rate of NADPH oxidation (Figures 10, 10a, 11, and 11a) from 239% to 323%, respectively, as shown in

Table III. However, the linear decrease in measured absorbance only held through the first 5 min. of measurement.

**Figure 9. Oxidation of 0.4 mM NADPH by Nitric Oxide Synthase in the presence of 333 μM $Ca(II)_3(3,5\text{-}DIPS)_6$ and 907 μM L-NMMA.**

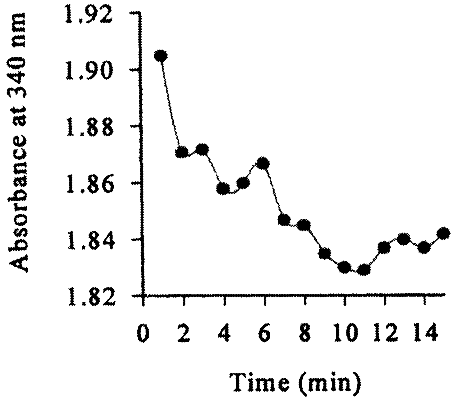

**Figure 9a. Initial rate for the oxidation of 0.4 mM NADPH by Nitric Oxide Synthase in the presence of 333 μM $Ca(II)_3(3,5\text{-}DIPS)_6$ and 907 μM L-NMMA.**

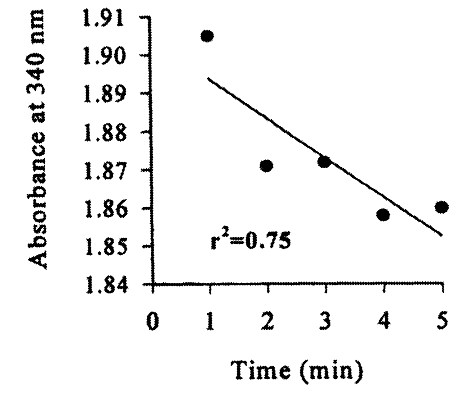

**Figure10. Oxidation of 0.4 mM NADPH by Nitric Oxide Synthase in the presence of 133 μM $Ca(II)_3(3,5\text{-}DIPS)_6$ and 113 μM L-NMMA.**

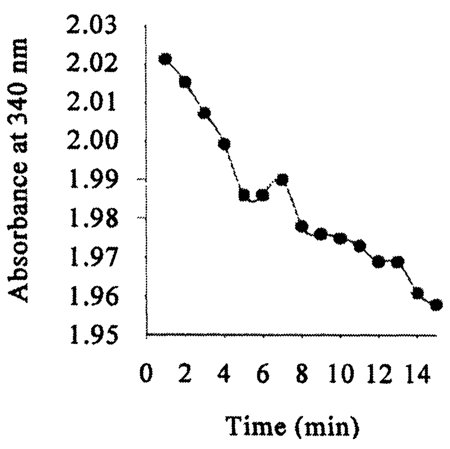

**Figure 10a. Initial rate for the oxidation of 0.4 mM NADPH by Nitric Oxide Synthase in the presence of 133 μM $Ca(II)_3(3,5\text{-}DIPS)_6$ and 113 μM L-NMMA.**

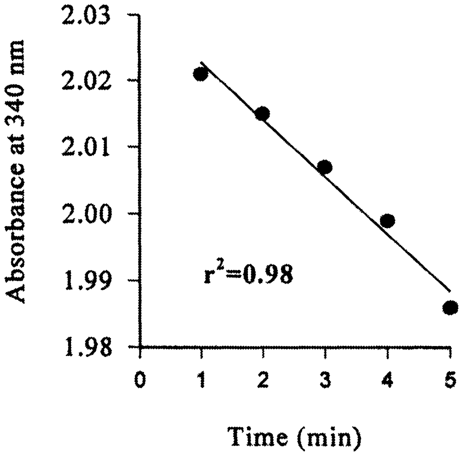

With a further increase in addition of $Ca(II)_3(3,5\text{-}DIPS)_6$ to 533 μM, there was a dramatic change in initial rate of NADPH oxidation (Figures 12 and 12a) to ˜27 % (Table III) of the oxidation of NADPH compared to the oxidation of NADPH in the absence of added $CaCl_2$ or only 22 % of the initial rate of NADPH oxidation in the presence of 133 μM $Ca(II)_3(3,5\text{-}DIPS)_6$. This decrease in initial rate of NADPH oxidation appears to be due to an oxidation-reduction cycling of NADPH until the NADPH was eventually consumed.

This oxidation-reduction equilibrium rationale is consistent with the observed poor fit of data to the fitted plot, $r^2 = 0.01$. This rationale is also consistent with the lack of smooth plots for the decrease in absorbance versus time observed for these reaction mixtures, which is most pronounced for the addition of 533 μM $Ca(II)_3(3,5\text{-}DIPS)_6$ (Figures 12).

Figure 11. Oxidation of 0.4 mM NADPH by Nitric Oxide Synthase in the presence of 267 μM Ca(II)$_3$(3,5-DIPS)$_6$ and 113 μM L-NMMA.

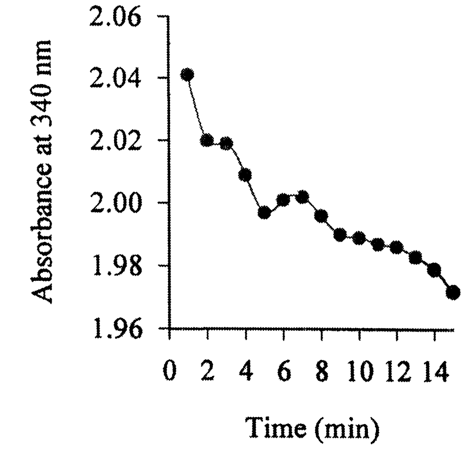

Figure 11a. Initial rate for the oxidation of 0.4 mM NADPH by Nitric Oxide Synthase in the presence of 267 μM Ca(II)$_3$(3,5-DIPS)$_6$ and 113 μM L-NMMA.

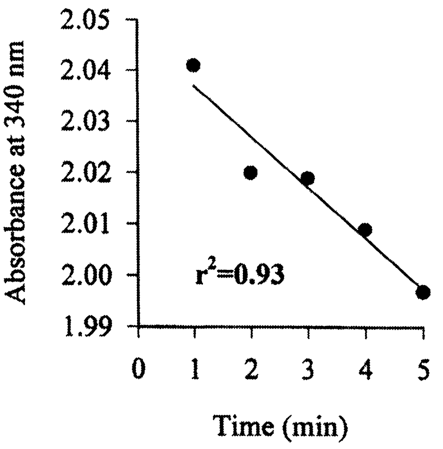

Figure 12. Oxidation of 0.4 mM NADPH by Nitric Oxide Synthase in the presence of 533 μM Ca(II)$_3$(3,5-DIPS)$_6$ and 113 μM L-NMMA.

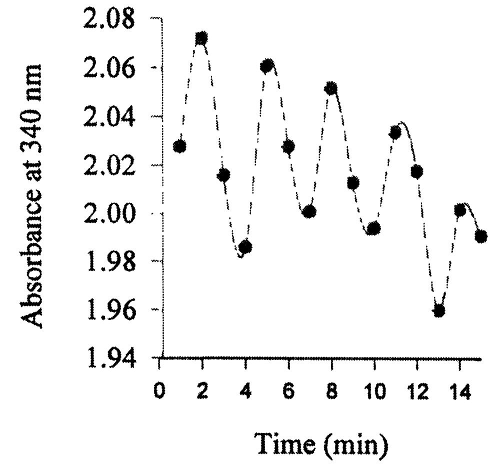

Figure 12a. Initial rate for the oxidatio of 0.4 mM NADPH by Nitric Oxide Synthase in the presence of 533 μM Ca(II)$_3$(3,5-DIPS)$_6$ and 113 μM L-NMMA.

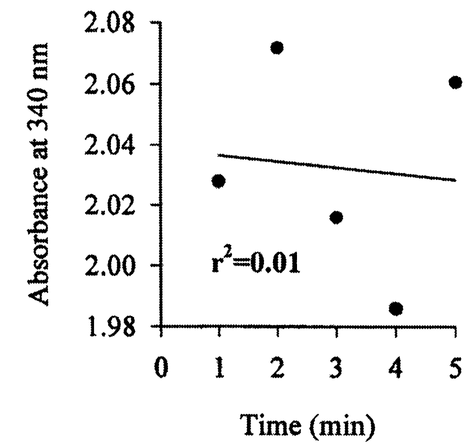

Discussion

Calcium(II)$_3$(3,5-DIPS)$_6$, a lipophilic Ca complex, failed to serve as a source of Ca for platelet activation in the presence of Thrombin since a 1500-fold increase in Ca concentration in this form compared to $CaCl_2$ failed to facilitate Thrombin-mediated aggregation. Calcium Chloride, a hydrophilic form of Ca, is required for platelet activation and aggregation in the presence of Thrombin.

Results presented in Figure 1 show that as increasing concentrations of Ca(II)$_3$(3,5-DIPS)$_6$ are added to the activated platelet system, the initial rate of aggregation is decreased or the addition of Ca(II)$_3$(3,5-DIPS)$_6$ to the activated platelet system caused a decrease in initial rate of aggregation, an increase in percent inhibition of aggregation.

Interestingly, the addition of L-NMMA caused an increase in activated platelet aggregation consistent with the suppression of endogenous platelet NOS synthesis of NO. Adding both L-NMMA and Ca(II)$_3$(3,5-DIPS)$_6$ to the activated platelet system dramatically decreased the rate of activated platelet aggregation, overcoming the increase in aggregation due to the addition of L-NMMA alone, which is most likely due to the activation of NOS by Ca(II)$_3$(3,5-DIPS)$_6$ and synthesis of NO. This may be interpreted as activation of Calmodulin, which in turn activates NOS.

This inhibition may be due to the passive translocation of $Ca(II)_3(3,5\text{-DIPS})_6$ across platelet plasma membrane and bonding of Ca via ligand exchange to activate Calmodulin phosphodiesterase activity which in turn activates NOS leading to the synthesis of NO and inhibition of platelet aggregation.

To examine the mechanism of $Ca(II)_3(3,5\text{-DIPS})_6$ activation of NOS, there was a need to determine whether or not this Ca complex serves as a bioavailable form of Ca in activating NOS. Results presented in Figure 2 show that the addition of NOS to the incomplete reaction mixture containing no added Ca caused a time related decrease in absorbance at 340 nm due to the oxidation of NADPH. Because NOS cannot be activated without Ca and therefore cannot oxidize NADPH in the absence of Ca, the purchased Calmodulin must already be nearly fully Ca activated. As shown in Table 2, the addition of $CaCl_2$ caused only a small increase in rate of NADPH oxidation by NOS. The addition of 333 µM $Ca(II)_3(3,5\text{-DIPS})_6$ to the incomplete reaction mixture, which represents the addition of the same molar concentration of Ca as in 0.9 mM $CaCl_2$ caused an additional increase in initial rate of NADPH oxidation by NOS in comparison with the complete reaction mixture containing 0.9 mM $CaCl_2$.

According to previous reports [2 and references therein], L-NMMA inhibits the activity of NOS. Results presented in Figures 5 to 10 and Table 2 are inconsistent with these reports. As increasing concentrations of L-NMMA were added to the reaction mixture containing 0.9 mM $CaCl_2$, the rate of NADPH oxidation increased (Figures 5, 5a, 6, 6a, 7, and 7a). These results suggest that L-NMMA may not always inhibit NOS activity and that L-NMMA actually serves as a oxidizable substrate for NOS which leads to an increase in the consumption of NADPH. This is consistent with the report by Hecker et al. [5] that L-NMMA led to an increase in oxidation of L-Arginine and L-Citrulline synthesis in cultured bovine endothelial cells in explaining why L-NMMA was less effective than L-$N^G$-nitroarginine methyl ester (L-NAME) in inhibiting rabbit aortic ring relaxation and in increasing blood pressure in anesthetized rats. It was suggested that a novel deaminase, a $N^G$,-$N^G$-dimethylarginine dimethylaminohydrolase, might be used to account for the conversion of L-NMMA to L-Citrulline, which was then metabolically converted to L-Arginine. In addition to this plausible accounting for increased NO synthesis with the addition of L-NMMA, it is also plausible that the P-450-mimetic activity suggested for NOS [6] might convert L-NMMA to L-Arginine and formaldehyde. This P-450-mimetic activity and the increased concentration of L-Arginine would account for the increased rate of NADPH consumption in the presence of increasing concentrations of L-NMMA. This increase in consumption of NADPH is also consistent with the reported failure of L-NMMA to inhibit the reduction of Cytochrome C by purified pituitary cell NOS [6]. We suggest that L-NMMA may serve as a substrate for NOS and that reducing equivalents derived from NADPH reduce Fe(III) of NOS to Fe(II) which in turn may activate oxygen for incorporation into L-NMMA to yield L-Arginine and formaldehyde. It may also be possible to convert L-NMMA to $N^G$-monomethyL-L-citrulline (L-NMMC) and NO by NOS in pituitary cells. Additional studies are required to examine the possibility that L-NMMA is converted to L-Arginine and formaldehyde or to L-NMMC and NO with this *in vitro* system.

The reaction system containing 907 µM L-NMMA and 333 µM $Ca(II)_3(3,5\text{-DIPS})_6$ caused NOS to oxidize NADPH at a faster initial rate than the system containing 0.9 mM $CaCl_2$ (Figures 7, 7a, 8, 8a). This demonstrates that $Ca(II)_3(3,5\text{-DIPS})_6$ is a better or more bioavailable source of Ca than $CaCl_2$ for the activation of NOS. As shown in Table 2, the initial rate of NADPH oxidation in the reaction mixture containing 113 µM L-NMMA increased as the concentration of $Ca(II)_3(3,5\text{-DIPS})_6$ was increased from 113 µM to 267 µM (Figures 9, 9a, 10, and 10a). These initial rates are faster than the initial rate observed for the reaction mixture containing 0.9 mM $CaCl_2$ and 113 µM L-NMMA, which further supports the possibility that $Ca(II)_3(3,5\text{-DIPS})_6$ serves as a more bioavailable form of Ca for NOS activation. The initial rate of NADPH oxidation for the system containing 533 µM $Ca(II)_3(3,5\text{-DIPS})_6$ (Figures 12 and 12a) underwent a dramatic decrease in comparison with the initial rate observed for the addition of 267 µM $Ca(II)_3(3,5\text{-DIPS})_6$ (Figures 11 and 11a). The addition of this very large concentration of $Ca(II)_3(3,5\text{-DIPS})_6$ appears to have allowed the oxidation of NADPH to NADP, however NADP is then reversibly reduced to NADPH which is then oxidized to NADP again in a cyclic process until the NADPH is consumed. Further studies of this system are needed to determine the mechanism of this cyclic oxidation-reduction process which may be an interesting biochemical redox characteristic of the NOS enzyme system.

Since Ca(II)$_3$(3,5-DIPS)$_6$ inhibited the aggregation of platelets and overcame the aggregation effect of L-NMMA, it is concluded that Ca(II)$_3$(3,5-DIPS)$_6$ activated Calmodulin, which in turn activated NOS leading to the synthesis of NO, and inhibition of platelet aggregation. The blockade of the effect of L-NMMA by the addition of Ca(II)$_3$(3,5-DIPS)$_6$ supports this conclusion.

Demonstrating that Ca(II)$_3$(3,5-DIPS)$_6$ increases the rate of NADPH oxidation by NOS offers support for the suggestion that L-Arginine is converted to L-Citrulline and NO, a mechanism consistent with the observation that Ca(II)$_3$(3,5-DIPS)$_6$ inhibits platelet aggregation.

Since the addition of Ca(II)$_3$(3,5-DIPS)$_6$ caused an increase in initial rate of NADPH oxidation in comparison with the initial rate observed for the complete reaction mixture containing equimolar $CaCl_2$, it is concluded that Ca(II)$_3$(3,5-DIPS)$_6$ is a more bioavailable form of Ca in activating Calmodulin, which in turn activates NOS.

Results obtained in these studies demonstrate that L-NMMA does not inhibit the activity of NOS in this *in vitro* system, but actually serves as a substrate for NOS and increases the rate of oxidation of NADPH by NOS. Although L-NMMA has been reported to be a NOS inhibitor in *in vivo* systems, it may act as a substrate for NOS in *in vitro* systems. Since L-NMMA serves as a oxidizable substrate for NOS, the conversion of L-Arginine to L-Citrulline and NO by NOS may be impeded *in vivo* if the rate of oxidation of L-NMMA to L-NMMC and NO is slower than the rate of conversion of L-Arginine to L-Citrulline and NO or L-NMMA simply serves as a reducible substrate for NOS and this consumption of reducing equivalents serves to decrease the conversion of L-Arginine to L-Citrulline and NO. Further studies of L-NMMA metabolism by NOS *in vitro* should include measuring the rate of NADPH oxidation in the presence of L-NMMA alone, without L-Arginine, and measuring the formation of NO using the Greiss test for metabolites of NO, nitrite and nitrate, and the measurement of L-Citrulline.

Results of the present studies suggest that Ca(II)$_3$(3,5-DIPS)$_6$ may be useful in facilitating activation of NOS and enabling NO-dependent physiological processes such as neuronal transmission, consciousness, dilation of blood vessels, leukocyte destruction of invading pathogens and cancer cells, leukocyte responses in inflammation, pain perception, and decreasing platelet aggregation. Decreasing platelet aggregation with Ca(II)$_3$(3,5-DIPS)$_6$ may be a particularly useful approach to preventing or treating cerebral and myocardial infarction and atherosclerosis consistent with the suggestion of Buechler et al.[7].

Finally, this new spectrophotometric method for the study of NOS enzyme activity may also be useful in examining other essential or non-essential metalloelement complexes for their potential effects on NOS inhibition, modulation, or down-regulation.

Acknowledgments

We are indebted to Professor Philip R. Mayeux for his assistance in enabling our activated platelet studies.

References

1. J. A. Lancaster, Jr., *American Scientist* May-June, **80** (1992) 248.
2. S. Moncada, R. M. J. Palmer, E. A Higgs, *Pharmacol. Rev.***43** (1991) 109.
3. R. W. Colman, *Proc. Soc. Exptl. Biol. Med.***197** (1991) 242.
4. M. W. Radomski, T. Zakar, E. Salas, *Meth. Enzymol.* **269** (1996) 88.
5. M. Hecker, J. A. Mitchell, H. J. Harris, M. Katsura, C. Thiemermann, J. R. Vane, *Biochem. Biophysics. Res. Comm.* **167** (1990) 1037.
6. D. J. Wolff, and G. A. Datto., *Biochem. J.* **285** (1992) 201.
7. W, A Buechler, K. Ivanova, G. Wolfram, C. Drummer, J.-M. Heim, R. Gerzer, *Ann. N. Y. Acad. Sci.* **714** (1994) 151.

**Received: November 22, 1999 - Accepted: February 12, 2000 -**
**Received in revised camera-ready format: February 15, 2000**

